# Swine enteric colibacillosis: diagnosis, therapy and antimicrobial resistance

**DOI:** 10.1186/s40813-017-0063-4

**Published:** 2017-08-08

**Authors:** Andrea Luppi

**Affiliations:** Istituto Zooprofilattico Sperimentale della Lombardia e dell’Emilia Romagna (IZSLER), Brescia, Italy

**Keywords:** Colibacillosis, ETEC, Pig, Diarrhoea, Diagnosis, Control

## Abstract

Intestinal infection with enterotoxigenic *Escherichia coli* (ETEC) is an important disease in swine resulting in significant economic losses. Knowledge about the epidemiology, the diagnostic approach and methods of control are of fundamental importance to tackle the disease. The ETEC causing neonatal colibacillosis mostly carry the fimbriae F4 (k88), F5 (k99), F6 (987P) or F41, while the ETEC of post-weaning diarrhoea carry the fimbriae F4 (k88) and F18. These fimbriae adhere to specific receptors on porcine intestinal brush border epithelial cells (enterocytes), starting the process of enteric infection. After this colonization, the bacteria produce one or more enterotoxins inducing diarrhoea, such as the heat stable toxin a (STa), the heat stable toxin b (STb), and the heat labile toxin (LT). A role in the pathogenesis of the disease was demonstrated for these toxins. The diagnosis of enteric colibacillosis is based on the isolation and quantification of the pathogenic *E.coli* coupled with the demonstration by PCR of the genes encoding for virulence factors (fimbriae and toxins). The diagnostic approach to enteric colibacillosis must consider the differential diagnosis and the potential different causes that can be involved in the outbreak.

Among the different methods of control of colibacillosis, the use of antimicrobials is widely practiced and antibiotics are used in two main ways: as prophylactic or metaphylactic treatment to prevent disease and for therapeutic purposes to treat diseased pigs.

An accurate diagnosis of enteric colibacillosis needs an appropriate sampling for the isolation and quantification of the ETEC responsible for the outbreak by using semi-quantitative bacteriology. Definitive diagnosis is based on the presence of characteristic lesions and results of bacteriology along with confirmation of appropriate virulence factors to identify the isolated *E.coli*. It is important to confirm the diagnosis and to perform antimicrobial sensitivity tests because antimicrobial sensitivity varies greatly among *E. coli* isolates. Growing concern on the increase of antimicrobial resistance force a more rational use of antibiotics and this can be achieved through a correct understanding of the issues related to antibiotic therapy and to the use of antibiotics by both practitioners and farmers.

## Background

*Escherichia coli* is a gram negative peritrichously flagellated bacteria belonging to the family Enterobatteriaceae and is the causative agent of a wide range of diseases in pigs, including neonatal diarrhoea and post-weaning diarrhoea (PWD), which are important causes of death occurring worldwide in suckling and weaned pigs respectively [1].

**Fig. 1:**
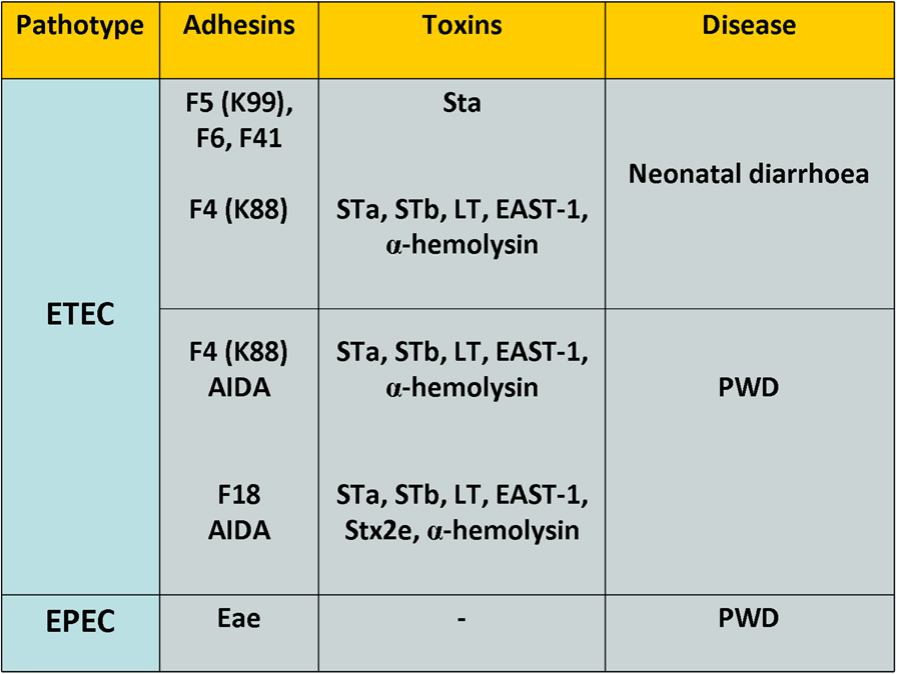
Pathotypes, adhesins and toxins of porcine pathogenic *E.coli* responsible for neonatal and post-weaning colibacillosis (AIDA: Adhesin involved in diffuse adherence; EAST-1: Enteroaggregative heat stable enterotoxin)

Two main pathotypes are involved in enteric colibacillosis: enterotoxigenic *E.coli* (ETEC) and enteropathogenic *E.coli* (EPEC). ETEC is the most important pathotype in swine and include different virotypes (this term is used to describe strains characterized by different combinations of toxins and fimbriae). Outbreaks of neonatal and post-weaning diarrhoea due to ETEC infection, generally affecting a high proportion of pigs, are often recurrent in the same herds and require expensive control measures. Enteric colibacillosis may result in significant economic losses due to mortality, decreased weight gain, cost for treatments, vaccinations and feed supplements [1]. Depending on the severity of the disease, the cost of PWD was estimated to range from €40 to €314 per sow [2]. ETEC possess fimbriae which adhere to enterocytes and elaborate one or several enterotoxins (Fig. 1) that induce secretory diarrhoea, causing some of the most significant diseases in the pig industry worldwide, such as neonatal colibacillosis and PWD.

Strategies commonly used to prevent and control neonatal colibacillosis should be aimed to reduce the number of pathogenic *E.coli* in the environment, implementing hygienic measures and internal and external biosecurity. The maintenance of suitable environmental conditions and piglets’ high level of immunity, guaranteed by lactogenic immunity and by vaccinations of sows against ETEC F4 (k88), F5 (k99), F6 (987P) and F41, reduce the risk of the disease’s development.

Various approaches have been used to prevent ETEC PWD, including passive administration with specific antibodies, dietary supplementation such as prebiotics and probiotics and dietary preventive measures, genetic breeding for ETEC-resistant herds and live oral nontoxigenic *E.coli* vaccines [3].

Even if some of the preventive approaches reported above for both neonatal and post-weaning colibacillosis have shown some promise and efficacy, antibiotics are still frequently used to treat enteric colibacillosis, administered by the parenteral and oral routes. Under-dosing is frequent with oral administration in pigs and this condition can favour the selection of resistant bacteria [4]. Antimicrobials commonly used to treat enteric colibacillosis must be chosen for their ability to achieve therapeutic concentrations in the intestinal content. The most frequently used are enrofloxacin, apramycin, ceftiofur, neomycin, gentamicin, amoxicillin/clavulanic acid, trimethoprim/sulphonamide and colistin [1]. Antimicrobial resistance to apramycin, neomycin, trimethoprim-sulfonimide and colistin has been increasingly observed, in particular in ETEC strains causing PWD [3].

Managing enteric colibacillosis in pigs requires an understanding of the pathotypes and the virotypes of *E.coli* involved and the conditions under which they are capable of causing disease, in order to implement appropriate diagnostics and strategies for prevention and control. The key element for approaching an outbreak of colibacillosis in order to reach a reliable and accurate diagnosis, is the knowledge of the diagnostic process and the interpretative criteria of diagnostic methods.

This paper is aimed at addressing some of the major questions that are frequently asked when faced with ETEC enteric neonatal and post-weaning colibacillosis in the field, which are the main subjects of this review, concerning the diagnostic approach and the interpretation of the specific investigations, the measures of control based on antibiotic therapy and the impact of antimicrobial resistance in the control of these diseases.

### Pathogenesis of pig enteric colibacillosis

#### Neonatal enteric colibacillosis

ETEC causing neonatal enteric colibacillosis enter the animal by ingestion and in the presence of predisposing environmental conditions and host factors, proliferate in the intestine and cause disease by means of specific virulence factors. The degree of colonization and proliferation determine whether or not disease results from infection. ETEC responsible for neonatal diarrhoea possess adhesins, surface proteins called fimbriae, identified as F4 (k88), F5 (k99), F6 (987P) and F41 (Fig. 1). The fimbriae allow the microorganism to adhere to specific receptors on the brush borders of the small intestine’s enterocytes. ETEC with the fimbriae F4 colonize the length of jejunum and ileum, while ETEC with fimbriae F5, F6, F41 mostly colonize the posterior jejunum and ileum [1]. Susceptibility to ETEC F5, F6 and F41 decreases with age and has been related to a reduction in the number of active receptors present on the intestinal epithelial cells with age. Most ETEC strains of neonatal colibacillosis produce heat stable enterotoxin STa, which binds guanylyl cyclase C glycoprotein receptor on the brush border of villous and crypt intestinal epithelial cells, stimulating the production of cyclic guanosine monophosphate (cGMP) leading to electrolyte end fluid secretion [1]. Excessive secretion leads to dehydration and eventual death [1]. Metabolic acidosis, defined as a state of decreased systemic pH, is a severe complication of neonatal colibacillosis and is due to lactate production. Most of the clinical signs that were formerly attributed to acidosis were in fact due to elevated blood levels of D-lactate. The source of D-lactataemia is bacterial fermentation of undigested substrate that reaches the large intestine due to the damage to the small intestinal mucosal epithelium. Respiratory compensation of acidosis occurs by hyperventilation, but this mechanism falls short due to an inadequate bicarbonate buffer [5].

Based on the concentration and affinity of the STa receptors, the posterior jejunum appears to be the major site of hypersecretion in response to STa. In the development of neonatal enteric colibacillosis, passive immunity plays a very important role. In particular, most neonatal infections can be prevented by passive colostral and lactogenic immunity [6]. Because ETEC infections are non-invasive gastrointestinal infections, mucosal i.e. lactogenic immunity rather than colostral i.e. systemic immunity is important to fight the disease [6]. For this reason, the presence of high levels of IgA in the milk of sows vaccinated or exposed to the pathogenic *E.coli* present in the environment of the piglets are able to prevent the small intestine colonization by ETEC. Because maternal vaccines are applied parenterally, the vaccination success, in particular of gilts, depends largely on these animals’ previous mucosal exposure to ETEC. Piglets are more prone to disease if specific antibodies are absent from the sow’s milk or they do not have access to a sufficient amount of milk.

#### Post-weaning enteric colibacillosis

As described for neonatal colibacillosis, *E.coli* causing PWD enter the animal by ingestion and in the presence of appropriate predisposing environmental conditions and host factors, proliferate in the intestine and cause disease by means of specific virulence factors [1].

Post-weaning ETEC strains mostly possess fimbriae F4 and F18, with some rare exceptions. Both fimbrial types (F4 and F18) have several variant subtypes based on antigenic differences. F4 variants ab, ac and ad have been described, even if almost all strains isolated from cases of PWD belong to the F4 ac subtype. F18 has two known variants, ab and ac. F18 ab is commonly associated with oedema disease (OD) strains, while F18 ac with PWD strains [7].

A non-fimbrial adhesin identified as adhesin involved in diffuse adherence (AIDA) has been associated with ETEC strains recovered from weaned pigs with PWD and there is evidence that it is causatively involved in diarrhoea experimentally induced in colostrum-deprived new-born piglets with STb encoding *E. coli* [8]. However, the role of EAST1 and AIDA in colibacillosis in pigs remains to be elucidated [9]. Post-weaning ETEC strains produce one or more of the known following enterotoxins: heat stable enterotoxins STa, STb, the heat-labile enterotoxin LT and the Enteroaggregative *E.coli* heat-stable enterotoxin (EAST1) [1]. The mechanism of action of STa has been described for neonatal colibacillosis. STb does not alter cGMP as described for STa, showing a different mechanism of action. Binding of STb to its receptor leads to an uptake of Ca^2+^ into the cells inducing the duodenal and jejunal secretion of water and electrolytes. In vivo significant accumulation of Na + and Cl − occur intraluminally following STb intoxication. In addition, STb stimulates bicarbonate (HCO3−) secretion [10].

LT is part of an important group of toxins - the AB5 toxin family. Two subtypes of LT, LTI and LTII have been described. Differences between LTI and LTII are largely due to dissimilarity in their B subunit. LTI can be divided in LTIh and LTIp, produced respectively by human and porcine ETEC. Strains expressing LT have also been shown to have an advantage in colonization promoting the adherence of ETEC in vitro and in vivo [10, 11]. LT permanently activates adenyl cyclase in the cell’s basolateral border and leads to hypersecretion of electrolytes and water [10] causing to dehydration. Metabolic acidosis is a complication of post-weaning colibacillosis, but is limited until circulatory collapse occurs.

EAST1 was reported in ETEC isolated from pigs with diarrhoea, however its role in the development of diarrhoea has not been elucidated [9].

#### Intestinal microbiota and etec

Environmental and maternal bacteria quickly colonize offspring gut after birth and shape theonset of a healthy intestinal immune system and its future development [12]. Intestinal microbiota is characterised by its high population density, extensive diversity, and complexity of interactions throughout the gastrointestinal tract [13]. Studies on the characterisation of the intestinal microbiota show that the major bacterial groups isolated from the pig intestine are *Streptococcus*, *Lactobacillus*, *Prevotella*, *Selenomona*, *Mitsuokella*, *Megasphera*, *Clostridia*, *Eubacteria*, *Bacteroides*, *Fusobacteria*, *Acidodaminococci*, and *Enterobacteria* [13]. Interestingly, it was reported that there is clear evidence that gut microbiota play an important role in driving host metabolism and that the diversity of the faecal bacterial community and their changes over time were different in pigs depending on their subsequent susceptibility to post-weaning diarrhoea [12]. The stomach and proximal small intestine (duodenum) contain relatively low numbers of bacteria (10^3^–10^5^ bacteria/g or ml of contents) due to low pH and/or rapid digesta flow. In contrast, the distal small intestine harbours a more diverse and numerically greater (10^8^ bacteria/g or ml of contents) bacterial population [13].

The mean number of *E.coli* biochemical phenotypes in piglets increased as animals aged [14] and *E. coli* populations in the pig faecal microbiota and in the farm environment are dynamic and show high levels of diversity [15].

Clinical manifestations of enteric colibacillosis obviously require the presence of pathogenic *E.coli* but also environmental changes and recognized risk factors [16]. Moredo et al. [17] demonstrated that the percentage of ETEC positive non-diarrhoeic pigs was 16.6% during the lactation period, 66% in the nursery phase and 17.3% in the finisher population. These data demonstrated that these pathogens can also be shed in faeces from healthy animals as already reported by Osek, in 1999 [18].

The barrier functions of the gastrointestinal tract in the neonatal piglet is not as developed as in mature animals due to the higher pH in the stomach, the lower proteolytic capacity, and the dependence on passive immune protection due to immunological immaturity [19]. The regeneration time of the small intestinal epithelium in day-old piglets is reported to be 7–10 days, as compared to 2–4 days in 3-week-old pigs. This difference is probably contributing to the susceptibility of infectious enteritis in new-born piglets, as a rapid turnover of enterocytes is considered a defence mechanism by the expulsion of infected cells [20]. Taken together, all of these conditions combined with predisposing factors, contribute to the neonatal piglet’s vulnerability to ETEC enteric infections.

After weaning, the change in the intestinal environment of piglets, mainly due to dietary changes, results in an alteration of the composition of the indigenous flora. The diversity of *E.coli* strains of intestinal flora is usually high in healthy pigs [15], while in enteric colibacillosis we observe an alteration of the balance between the bacteria present in the normal intestinal flora [14]. This condition leads to the proliferation of a dominating pathogenic strain, which colonizes the small intestine [21], rapidly reaching massive numbers to the order of 10^9^/g of contents. This is the reason why frequently, if not always, samples collected in diarrhoeic pigs affected by colibacillosis allow the isolation of a pure culture of pathogenic *E.coli*.

This information must be considered for a correct interpretation of diagnostic results. In particular, the evaluation of diagnostic findings should be made only in consideration of both clinical signs and pathological lesions, while also taking into account the number of isolated pathogenic *E.coli* strains belonging to the identified pathotype and virotype.

#### The diagnostic approach

The diagnosis of neonatal and post-weaning enteric colibacillosis includes the combination of different diagnostic procedures, starting from the observation of clinical signs and gross lesions, followed by appropriate bacteriological investigations and typing of the isolated bacterial strains.

#### Clinical signs, gross lesions and sampling

Neonatal diarrhoea due to enterotoxigenic *E.coli* is observed most commonly in piglets aged from 0 to 4 days of life, and in general, in an endemic condition, litters from first-parity sows could be more involved due to a lack of protection by passive immunity.

PWD due to *E.coli* is commonly observed 2–3 weeks after weaning and although not exceptionally, it can be recorded at 6–8 weeks after weaning.

When ETEC sustains neonatal diarrhoea, large quantities of watery to a creamy consistency scour are observed, with a distinctive smell and often white to yellow in colour.

**Fig. 2:**
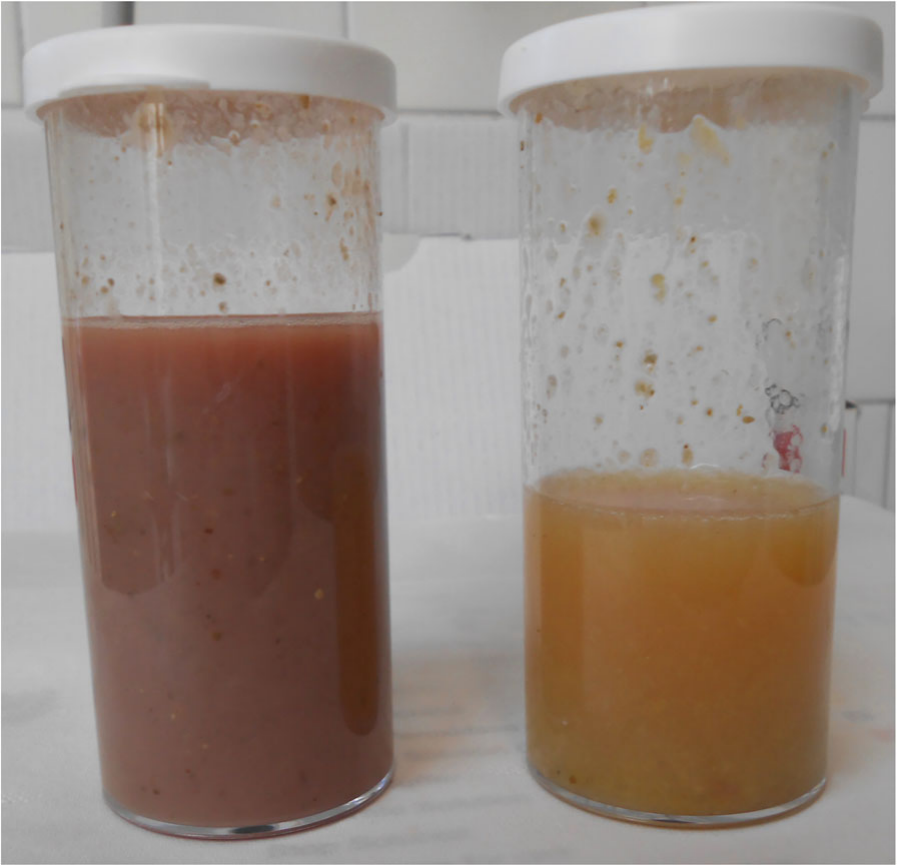
Diarrhoeic faeces of pigs suffering from ETEC F4 PWD

The cases of post-weaning colibacillosis due to ETEC are usually characterized by yellowish, grey or slightly pink watery diarrhoea with a characteristic smell, generally lasting one week (Fig. 2).

**Fig. 3:**
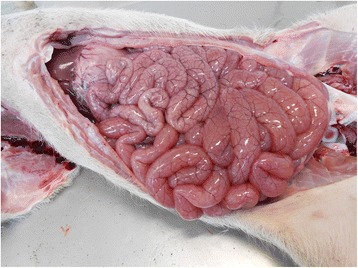
Intestine of a pig suffering from ETEC F4 PWD appears dilated, oedematous and hyperemic

**Fig. 4:**
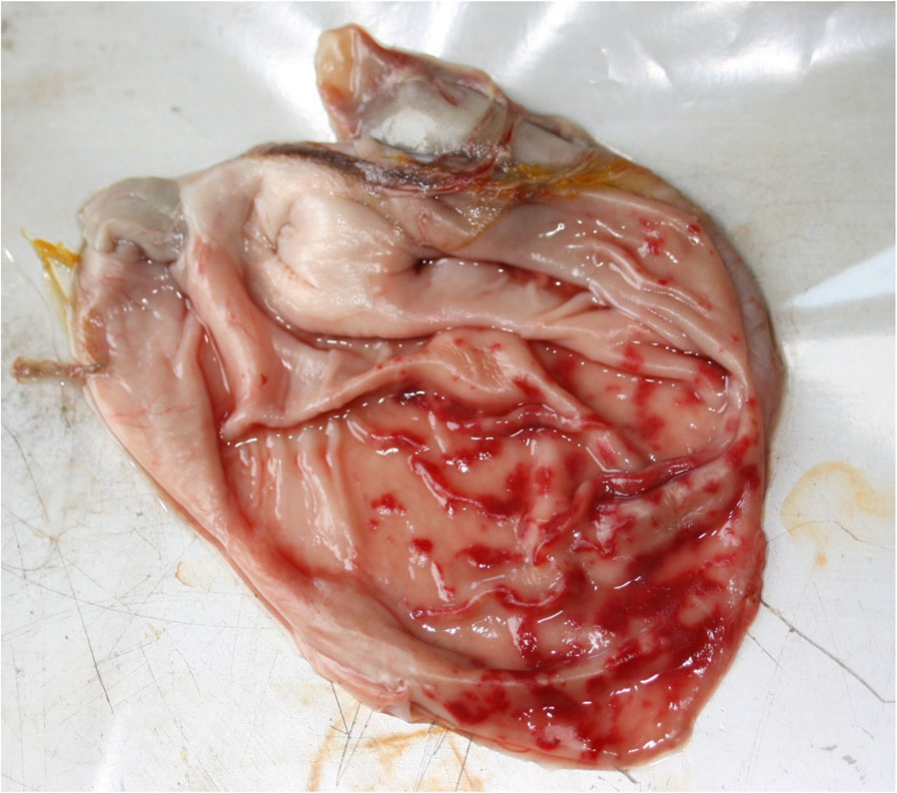
Stomach of a pig suffering from ETEC F4 PWD. The gastric fundus shows a severe hyperemia

Affected pigs are usually depressed with a reduced appetite and a rough sticky wet haircoat. Sudden deaths can occur, particularly at the start of the outbreak and dead pigs are usually dehydrated with sunken eyes. The small intestine is usually dilated, slightly oedematous and hyperaemic (Fig. 3). The stomach, usually dilated and full of clotted milk or dried feed, in neonatal or post weaning colibacillosis respectively, shows hyperaemia of the fundus (Fig. 4). The mesenteric lymph-nodes are enlarged and commonly hyperaemic. These lesions, even if not pathognomonic, are suggestive of enteric colibacillosis. For this reason the necropsy, both in the cases of neonatal and post-weaning colibacillosis, helps the pathologist in the choice of subsequent laboratory examination.

**Fig. 5:**
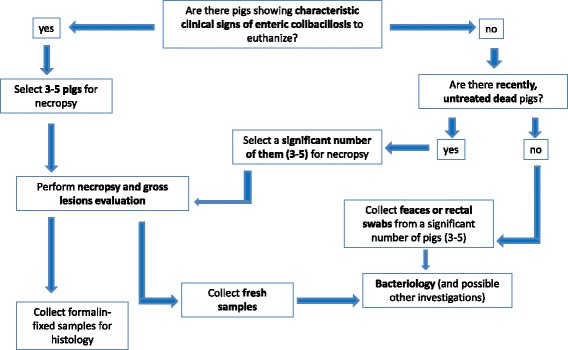
Flow chart for sampling of enteric colibacillosis (neonatal and post-weaning) in pigs

The most effective approach is to select a number of untreated pigs (3–5) suffering from diarrhoea for less than 12–24 h, and to humanely euthanize them and perform an accurate necropsy in order to evaluate gross lesions (evaluating the small intestine, colon, ileo-caecal valve, mesenteric lymph-nodes) and collect samples. Unopened segments of small intestine (in particular ileum and jejunum) and large intestine with the ends tied off should be taken and sent to the laboratory (fresh samples for bacteriological investigations and fixed in 10% buffered formalin for histology), with a bacteriological examination request for the isolation of the pathogenic *E. coli* strain involved in the outbreak, its quantification (pure culture or not), its typing and evaluation of the sensitivity to antibiotics (Fig. 5). Fresh samples should be stored at +4 °C and should arrive at the laboratory in less than 24 h.

Recent spontaneously dead animals can also be used for microbiological analysis. Since autolysis of the gut after death occurs promptly, tissues obtained from animals 4–6 h after death are usually not suitable for histopathological analyses. If there are no pigs showing characteristic clinical signs of enteric colibacillosis to euthanize or recently dead pigs available, it is advisable to collect faeces (directly from the animals and not from the soil) or rectal swabs from 3 to 5 pigs (Fig. 5).

**Fig. 6:**
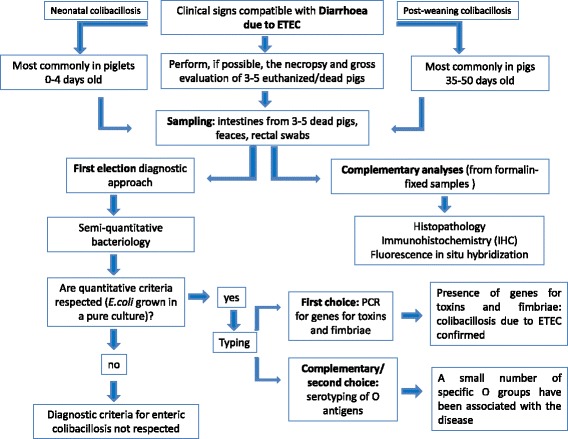
Diagnosis of enteric colibacillosis: proposed diagnostic tree and diagnostic criteria

The definitive diagnosis requires the combination of several investigations including quantitative bacteriology, the identification of virulence factors, usually by PCR, and histopathology as a complementary analysis, in order to have an integrated interpretation of the microscopic lesions observed with the pathogen detected. A diagnostic tree and diagnostic criteria that should be followed in the diagnosis of enteric colibacillosis is reported in Fig. 6.

#### Bacteriology and characterization of bacterial isolates

**Fig. 7:**
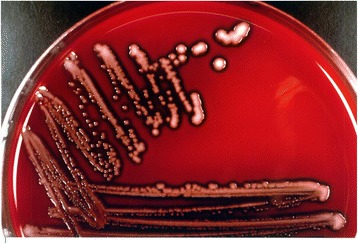
ETEC F4 isolated from the intestinal content of a pig suffering from PWD. The picture shows a pure culture of haemolytic *E.coli* on blood agar

The diagnosis of enteric colibacillosis is based on the bacteriological examination of samples of luminal content (first choice) or rectal swabs. The samples should be inoculated onto blood agar and McConckey agar or other media which are selective for Enterobatteriaceae such as Hektoen agar. These selective media allow differentiation of lactose fermenting (such as *E.coli*) from lactose non-fermenting Gram negative enteric bacilli. Colonies on solid media reach their full size within 1 day of incubation and vary from smooth to rough or mucoid. The characteristics of the colonies grown on blood agar and lactose fermentation on selective media give a first diagnostic indication. In particular, the presence of haemolytic colonies, both in neonatal diarrhoea and PWD, is often used as a rapid tool for the diagnosis of ETEC diarrhoea (Fig. 7). In general terms, ETEC isolated from cases of neonatal colibacillosis can appear as haemolytic (ETEC F4 positive) or non-haemolytic (ETEC F5, F6, F41) colonies on blood agar plates [1, 22]. ETEC isolated from cases of PWD are mostly haemolytic (ETEC F4 or F18) even if non-haemolytic strains can be observed. In a recent study, the authors reported that *E.coli* strains isolated from cases of PWD, and characterized as ETEC, were haemolytic in 97.6% of the cases. The remaining 2.4% non-haemolytic ETEC isolates, for which haemolytic activity was consistently tested, were recovered in France, Italy and Germany, and sharing the same virotype: F4, STa, STb [23].

The detection of pathogenic strains does not justify the disease in every case and it is important to consider that *E. coli* pathotypes can, of course, also be isolated from the gut habitat of healthy hosts as reported above.

Evaluation of diagnostic findings can therefore be made only in consideration of both the clinical and pathological observations, coupled with the quantification of the isolated pathogenic *E.coli*. For this reason, the high concentration of pathogenic *E.coli* in pure or nearly pure culture isolated from the small intestine (ileum and jejunum) are indicative of enteric colibacillosis. Since almost all ETEC F4 or F18 are haemolytic, the presence of a pure culture of haemolytic colonies can be used as a presumptive diagnosis of neonatal colibacillosis (ETEC F4) or post-weaning colibacillosis (ETEC F4 or F18) [1].

The interpretation of bacteriological negative results in animals treated with antibiotics is unreliable and requires repeating the examination with untreated pigs.

The identification of virulence genes encoding for the fimbriae and toxins of the isolated strain is crucial to ascertain its role in the clinical problem observed. Currently, in the diagnostic routine, genotypic analysis such as the polymerase chain reaction (PCR) for the detection of genes encoding for virulence factors is performed in many laboratories to characterize the isolated strains. Primers recognising genes encoding for toxins (STa, STb, LT and EAST1) and fimbriae (F4, F5, F6, F18, F41) of ETEC, for the outer membrane protein Eae or intimin in enteropathogenic *E.coli* (EPEC) and for Stx2e toxin in STEC (*E.coli* strains involved in oedema disease) strains, are available and can be used to perform PCR assays for daily routine diagnostics [24]. Interestingly, certain F18 strains produce both enterotoxins and the Stx2e toxin. These strains are classified ETEC rather than STEC, since they produce clinical PWD more than oedema disease [1].

The use of end point PCR for the direct identification of virulence factors in samples from diseased pigs, without performing a semi-quantitative bacteriology and typing of individual isolates, can make the interpretation difficult and unreliable. This diagnostic approach does not allow the quantification of the pathogen, and can give a mix of all the detectable virulence factors belonging to different *E.coli* strains present in the sample and, as a result, false combinations of these factors. In addition, it cannot be excluded that similar genes of virulence factors of other intestinal Enterobacteriaceae might be detected. As an example, a study performed on bacteria isolated from cases of diarrhoea in Children in Mexico showed the ST toxin gene of one strain identified as *M. morganii* being 100% identical to an ST toxin gene of *E. coli* [25]. The presence of genes encoding the LT toxin was previously reported in *M. morganii* obtained from stool samples of travelers with diarrhoea [26].

Development of quantitative PCR assays (qPCR) has become a feasible option for diagnosis [27] alone or combined with bacteriology. Ståhl and colleagues reported that the sensitivity of the qPCR was higher when compared to cultivation of *E. coli* F4 and *E. coli* F18 from faecal specimens from pigs with diarrhoea. In 34% of the samples that were positive in F4-qPCR and/or F18-qPCR, pathogenic *E. coli* were not detected by cultivation. When more than 10^7^ CFU/g of *E. coli* F4 and/or *E. coli* F18 were detected, this was correlated with the cultivation of a high number of potentially pathogenic *E. coli* [27]. Even if the quantification of the pathogenic *E.coli* using qPCR represents a promising diagnostic method for enteric colibacillosis, semi-quantitative bacteriology is of fundamental importance to perform the isolation and antimicrobial susceptibility testing of the *E.coli* strain responsible for the outbreak.

Usually, outbreaks of F4 positive *E.coli* tend to involve only one strain at any one time, even if mixed infections with the isolation of different virotypes in the same outbreak were observed. In these cases, one virotype probably predominates in any given outbreak [1]. For these reasons, it would be appropriate to type more isolates obtained from different pigs involved in the outbreak, after the quantitative bacteriological examination and compatibly with the costs for the examinations, in order to determine if more than one virotype is involved in an outbreak of enteric colibacillosis. As an example, it might be advisable to test samples from 5 representative pigs with diarrhoea and typing 3 isolates previously chosen for their cultural and biochemical characteristics. Although this approach does not give absolute results, it certainly increases the reliability of the results obtained.

**Fig. 8:**
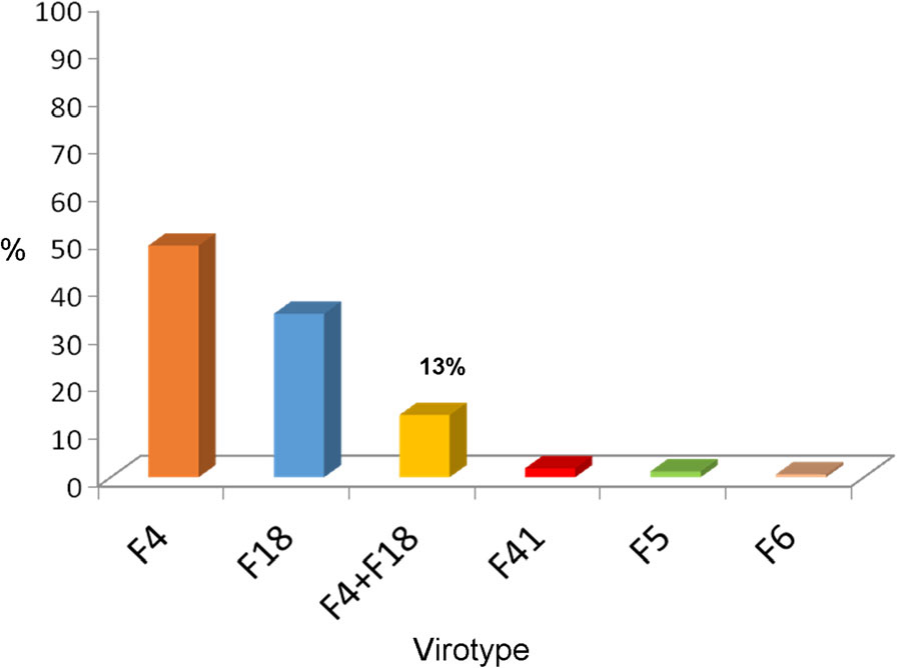
Different virotypes isolated from 160 cases of PWD in different European countries, sampling 5 pigs with diarrhoea and typing 3 isolates previously chosen for their cultural and biochemical characteristics for each outbreak (data not published)

A study performed on 160 European herds during PWD outbreaks, following the protocol of sampling reported above, showed that mixed infections (ETEC F4 and F18) were observed in 13% of the cases (data not published) (Fig. 8).

Pathogenic *E.coli* may be also identified by the serotyping of O antigens (cell wall LPS), since a small number of specific O groups have been associated with the disease (Table 1).

**Table 1:** O serogroups most frequently implicated as enterotoxigenic *E.coli* that cause neonatal diarrhoea in pigs

ETEC Adhesins	O serogroups	Disease
F5, F6, F41	O8, O9, O20, O64, O101	Neonatal diarrhoea
F4	O8, O138, O141, O145, O147, O149, O157	

A complete serotyping of H (flagellar protein antigen) and O antigens, with the additional identification of K antigens (capsular polysaccharide), is the standard method for the definition of all serotypes, but in general is carried out in few reference laboratories.

Different clones within a serogroup may have evolved acquiring different virulence genes, resulting in clonal variation associated with a particular region or country. As an example, ETEC of serogroup O139, associated worldwide with the F18ab fimbriae, typically cause PWD in Australia and OD in Europe, while the predominant serogroup associated with PWD in pigs worldwide is O149 [1] (Table 2).

**Table 2:** Common serovirotypes of pathogenic *E.coli* from pigs with PWD (modified from Fairbrother and Gyles [1])

Fimbrial adhesins	Serovirotypes
F4	O149:LT:STb:EAST-1
	O149:LT:STa:STb:EAST-1
	O149:LT:STb
F18	O149:LT:STb:EAST-1
	0138:STa:STb
	O138:LT:STb:EAST-1:Stx2e
	O139:Stx2e:(AIDA)
	O147: STa:STb:AIDA

### Histopathology

Histopathology in formalin-fixed, paraffin embedded tissues (ileum, jejunum and large intestine should be included) can be used as an additional investigation for a definitive diagnosis of colibacillosis. In piglets suffering from neonatal enteric colibacillosis, F4 positive ETEC are observed adhering to most of the jejunum and ileum’s enterocyte brush border membrane of intestinal mucosa, while other ETEC mainly colonize the distal jejunum or the ileum [1]. Other changes include vascular congestion, haemorrhages and an increased number of inflammatory cells (neutrophils and macrophages in the *lamina propria*) [1]. Microscopic lesions in ETEC PWD are characterized by bacterial layers observed in patches on the apical surface of villous epithelial cells in the ileum and less consistently in the jejunum. Mild villous atrophy and an increased number of neutrophils may be observed in the superficial *lamina propria* [1]. Immunohistochemistry and fluorescence in situ hybridization can be used as tools for the confirmation of the aetiology of the miscroscopic lesions observed.

Criteria and methods used for the identification of *E.coli* strains isolated from cases of neonatal and post-weaning colibacillosis have been reported in Table 3.

**Table 3:** Interpretative criteria used for the diagnosis of *E.coli* neonatal and post-weaning diarrhoea (modified from Fairbrother and Gyles, 2012) [1]

Criteria	ETEC		ETEC			EPEC
	F4	F18	F5	F6	F41	
Haemolytic colonies	Nearly all		None			None
Genotypic analysis	Fimbriae and toxins					Eae (intimin)
Serogroups (most prevalent)	O8, O138, O139, O141, O147, O149, O157		O8, O9, O20, O64, O101			O45, O103
Slide agglutination (F adhesin serotyping)	All	Not reliable	Not reliable			Not reliable
Histology	Bacterial layers are observed in patches on the apical surface of villous epithelial cells in the ileum and to a lesser extent in the jejunum					Multifocal “attaching and effacing” (AE) lesions involving the small intestine (duodenum, ileum, cecum)

### Differential diagnosis

Enteric disease outbreaks in pigs are frequently multifactorial. Focusing on the diagnosis and subsequent control strategies without take into account all the possible differential diagnoses or multiple agents involved can misguide practitioners. The diagnostic evaluation of neonatal and post-weaning colibacillosis requires similar standard diagnostic procedures. The diagnostic pathway is kept easy if the lesions observed during necropsy are strongly suggestive. This is the case in enteric colibacillosis, where bacteriology can usually easily confirm the suspect given by the recorded pathological lesions. Diarrhoea in the pre-weaned piglet is probably more straightforward to identify, treat, and prevent than post-weaning diarrhoea. In particular, ETEC neonatal diarrhoea must be differentiated from other causes of diarrhoea, such as *Clostridium difficile*, *Clostridium perfrigens* type A and C, enteric coronavirus (TGEV, PEDV) and rotavirus groups A, B and C. In piglets older than 7 days, coccidiosis due to *Isospora suis* should also be considered [28] (Table 4).

**Table 4:** Differential diagnosis of the main agents of neonatal diarrhoea (modified from Martelli et al. 2013) [28]

Disease/Etiological Agent	Age	Diarrhoea	Gross Lesions	Lethality	Laboratory diagnostic methods
Colibacillosis *E.coli* (ETEC)	Most commonly from 0 to 4 days	Yellowish, grey or slightly pink alkaline pH	Distension, congestion of small intestine. Stomach full of curdled milk	Can reach 70%	Culture/isolation. Typing of isolates usually by PCR Histopathology
Clostridiosis *C.perfrigens* type C	PA: 1 days A: 3 days SA: 7 days C: 10–14 days	PA: watery yellowish bloody A: brown bloody SA: watery grey/yellow C: yellow/grey	Jejunum and ileum mostly involved. Haemorrhagic enteritis Bloody ascitis	100% in PA and A forms	Culture/isolation. Typing/toxin identification. Histopathology
Clostridiosis *C.perfrigens* type A	Generally diarrhoea is observed within 48 h of birth	Mucoid, pink without blood	Jejunum and ileum mostly involved Pasty content Presence of necrotic membrane	Generally low if not complicated	Culture/isolation. Typing/toxin identification. Histopathology
Clostridiosis *Clostridium difficile*	In the first week of life	Pasty and yellow	Mesocolon oedema. Typhlocolitis with focal erosions	Variable. Up to 50%	Culture/isolation. Toxin identification
Coronavirus PEDV TGEV	All	Watery yellow/white/grey Watery yellow, white, grey, greenish; acid pH	Empty stomach. Small intestine was thinned and congested	Differs between strains and between naïve and endemic infected herds. Very high (80–100%) in suckling piglets belonging to naïve infected herds	PCR Histopathology Viral isolation
Rotaviral enteritis Rotavirus	From 1 to 5 weeks	Watery, sometime pasty. Acid pH	Small intestine was thinned. Milk in the stomach	Low (in endemic infected herds) <20%	PCR Histopathology Viral isolation
Coccidiosis *Isospora suis*	Not before 5 days. More frequent around 14 days	Yellow and pasty. Alkaline pH	Small intestine. Enteritis with fibrino-necrotic membrane	Very low or not observed	Microscopic evaluation after flotation

*PA* per-acute, *A* acute, *SA* sub-acute, *C* chronic

ETEC PWD should be differentiated from other causes of diarrhoea already described in piglets such as EPEC, enteric coronavirus (TGEV, PEDV), rotavirus groups A, B and C, salmonellosis, proliferative enteropathy due to *Lawsonia intracellularis* and *Brachyspira spp*. [28] (Table 5).

**Table 5:** Differential diagnosis of the main agents of post-weaning diarrhoea (modified from Martelli et al. 2013) [28]

Disease/Etiological Agent	Age	Diarrhoea	Gross Lesions	Lethality	Laboratory diagnostic methods
Colibacillosis *E.coli* (ETEC, EPEC)	Most commonly post-weaning until 45–50 days	Yellowish, grey or slightly pink alkaline pH	Distension, congestion of small intestine. Gastritis and stomach full of feed	Can reach 25%	Culture/isolation. Typing of isolates usually by PCR. Histopathology
Swine dysentery *Brachyspira hyodysenteriae*	Frequent in the growing-fattening periods	Muco-haemorrhagic	Muco-haemorrhagic and fibrino-necrotic typhlocolitis	Variable, usually low	Culture/isolation. Typing by PCR. Histopathology
Salmonellosis (*Salmonella typhimurium*)	Mostly in the growing-fattening periods	Yellowish, greenish, muco-haemorrhagic	Necrotic lesions yellowish membrane (small and large intestine); Prominent Payer patches	Low	Culture/isolation
PED and TGE Coronavirus PEDV TGEV	All	Watery yellow/white/grey Watery yellow, white, grey, greenish; acid pH	Empty stomach. Small intestine was thinned and congested	Can be high; less severe than in neonates	PCR Histopathology Viral isolation
Rotaviral enteritis Rotavirus	From 1 to 5 weeks	Watery, sometime pasty. Acid pH	Small intestine was thinned.	Low, <20%	PCR Histopathology Viral isolation
Proliferative enteropathy *Lawsonia intracellularis*	Post-weaning	A: haemorrhagic C: greenish	Ileitis	Low	PCR Histopathology

*A* acute, *C* chronic

### Treatments of enteric colibacillosis

#### Symptomatic treatment

The effect of diarrhoea in pigs affected by enteric colibacillosis is a loss of liquids that leads to the dehydration of the animals. The administration of saline solution and rehydration is essential in many cases [1]. Pigs represent a particular problem in rehydration, since the intravenous route is impractical, as is subcutaneous administration. Intraperitoneal injection can be used, but the volume which can be infused is limited, and uptake is uncertain [29]. Fluid therapy consisting in electrolyte replacement solutions containing glucose given orally, is used for the treatment of dehydration and metabolic acidosis in pigs affected by colibacillosis [1]. Studies in rats and clinical studies in children have shown that oral rehydration solutions with low osmolality promoted intestinal fluid absorption, with beneficial effects on the course of diarrhoea [30].

#### Zinc oxide

Feed containing between 2400 and 3000 ppm of zinc reduce diarrhoea, mortality and improve growth. For a long while, it was thought that zinc oxide must have an antibacterial effect, especially against *E. coli.* Several antimicrobial mechanisms of zinc oxide were proposed based on studies performed in vitro: 1) hydrogen peroxide, which is generated from the surface of zinc oxide, can penetrate through the cell membrane, produce some type of injury, and inhibit the growth of the cells; 2) the affinity between zinc oxide and bacterial cells is an important factor for antibacterial activity. Other investigators showed that zinc oxide reduced bacterial adherence of ETEC F4 and blocked bacterial invasion by preventing increased tight junction permeability and modulating cytokine gene expression [31].

Zinc is poorly absorbed, so it becomes highly concentrated in manure with implications in terms of environmental pollution. The therapeutic use of zinc is currently debated. In general terms, bacteria in animals may develop resistance to Zn as well as to other heavy metals such as Cu. Resistance genes to Zn are often located on plasmids, which may be transferable to other bacteria, intra- and inter-species. Exposure to trace metals may also contribute to antibiotic resistance, even in the absence of antibiotics themselves. Zn supplementation to animal feed may increase the proportion of multi-resistant *E. coli* in gut microbiota [32]. Several studies have focused attention on heavy metals used in animal farming and possible mechanisms that could promote the spread of antibiotic resistance via co-selection. One report associated zinc with methicillin-resistant *Staphylococcus aureus* (MRSA) CC398 in Denmark [33], concluding that zinc compounds may be partly implicated in the emergence of MRSA clones. The co-selection mechanisms include co-resistance and cross-resistance. Co-resistance is defined as the close proximity of two or more genetic elements encoding for resistances. Sulphonamide resistance, for example, would follow the co-resistance path. The cross-resistance evolves when an antibacterial agent attacks the same target, for instance efflux systems that simultaneously transport two or more types of antibacterial agents. An example of cross resistance could be done with tetracycline, as zinc resistant strains would also expel tetracycline using the same efflux system [34].

Recently, the Committee for Medicinal Products for Veterinary Use (CVMP) of the European Medicines Agency (EMA) concluded the referral procedure for veterinary medicinal products containing zinc oxide to be administered orally to food-producing species. The Committee adopted an opinion by consensus concluding that the benefits of zinc oxide for the prevention of diarrhoea in pigs do not outweigh the risks for the environment. The CVMP highlighted that there is a risk of co-selection for resistance associated with the use of zinc oxide, but at the present time, that risk is not quantifiable. The Committee therefore recommended the refusal of the granting of marketing authorisations and the withdrawal of existing marketing authorisations for veterinary medicinal products containing zinc oxide [35].

#### Antimicrobials

##### The treatment of enteric colibacillosis: general principles of antimicrobial therapy

Antimicrobial therapy is required in many cases of enteric colibacillosis, besides using approaches to avoid infectious agents and clinical diseases. Antimicrobial therapy must be selected which reaches therapeutic concentrations in the intestinal lumen, as observed for different classes of antibiotics: β-lactam antibiotics (amoxicillin and the combination containing amoxicillin/clavulanic acid), cephalosporins (ceftiofur, cefquinome), aminoglucosides (apramycin, neomycin, gentamycin), aminocyclitols (spectinomycin) sulphonamide combined with trimethoprim (such as trimethoprim/sulphametoxazole), fluorochinolones (enrofloxacin, marbofloxacin and danofloxacin), quinolones (flumequine) and polymyxins (colistin sulphate) [1, 36].

The therapeutic approach and, consequently, the choice of the antibiotic for the treatment must consider several aspects:

1. The infection is located mainly in the small intestine. The antibiotic selected for the therapy must reach sufficient concentrations in the small intestine.
2. Empiric treatments are performed on the basis of knowledge on the individual herd and local data on the resistance pattern.
3. In many cases the evaluation of the isolated strain’s antimicrobial susceptibility is fundamental for a correct therapy.

An outbreak of colibacillosis frequently requires quick actions and therefore the use of antibiotics almost always precedes the results of the resistance pattern. As a result, in most cases the right choice of antibiotics remains the practitioner’s responsibility, for which the laboratory gives a retrospective result. The most basic information that the laboratory can provide is qualitative susceptibility results (the bacterial strain is susceptible, intermediate, or resistant to a specific antibiotic). Quantitative results obtained by the minimal inhibitory concentration (MIC) may be more useful than the traditional qualitative results, because MIC data define the degree of the pathogen’s susceptibility more precisely.

**Fig. 9:**
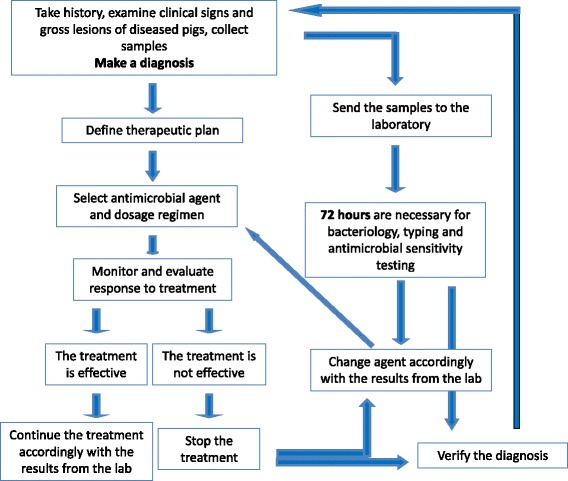
Steps in the initiation, management and reassessment of antibiotic therapy in an outbreak of enteric colibacillosis (Modified from Page and Gautier [40])

Figure 9 reports, as an example, the steps in the initiation, management and reassessment of antibiotic therapy in an outbreak of enteric colibacillosis.

Some European Countries, such as the Netherlands, introduced a classification of all antibiotics into first, second and third choice (last resort). This kind of approach was described by Burch et al. [37]. These classifications are continuously under revision. In general terms, first choice antibiotics can be used after a clinical diagnosis (empirical treatment), while second-choice antibiotics should be reserved for cases where sensitivity testing or clinical results has proven first-choice antibiotics are not effective, and third-choice antibiotics (such as third and fourth generation cephalosporins, fluorochinolones and macrolides) being reserved as last-resort antimicrobials, if no other options are available.

The antibiotic should be administered to all animals showing clinical signs referable to colibacillosis and sick pigs must be treated parenterally, since they eat and drink very little. In practice when mortality occurs, a metaphylactic approach is applied wherein all animals in the pens are treated where mortality has been observed. Guidelines for the prudent use of antimicrobials in veterinary medicine (2015/C 299/04) published on the official journal of the European Union considered the use of metaphylaxis and stated that antimicrobial metaphylaxis should be prescribed only when there is a real need for treatment and that the veterinarian should justify and document the treatment on the basis of clinical findings on the development of a disease in a herd or flock [38].

##### Pharmacokinetic/pharmacodynamic indices: general information on antimicrobial drugs used in the treatment of enteric colibacillosis

The efficacy of antimicrobial therapy is dependent upon the pathogen’s ability to respond to antimicrobial therapy, the drug exposure characteristics necessary to elicit the targeted microbiological response, and the ability to achieve the necessary active drug concentrations at the site of infection [39]. The relationship between systemic drug exposure and its corresponding clinical and microbiological effects is termed pharmacokinetics/pharmacodynamics (PK/PD) (Table 6). This PK/PD relationship, in turn, dictates the dose, dosing frequency, and duration of drug administration necessary to achieve the desired clinical and microbiological outcome. The PK component describes the handling of the drug by the host (absorption, distribution, metabolism, and elimination) (Table 7). The PD component describes the effect of the drug over time on the bacteria at the site of infection. Thus, the interplay between PK and PD reflects the relationship between the fluctuating concentrations of biologically active drug at the site of infection, as reflected by its serum or plasma drug concentrations, versus its effects on the targeted microbial pathogen [39].

**Table 6:** Classification of antibacterial drugs frequently used in the treatment of enteric colibacillosis according to pharmacokinetics and pharmacodynamics indices (modified from Ahmad et al. [41])

Drugs	Bacterial effect	PK/PD parameters
Fluoroquinolones (enrofloxacin)	Concentration dependent	Cmax/MIC and AUC/MIC
Cephalosporins (ceftiofur)	Time dependent	T > MIC
Sulphonamides + Diaminopyrimidines (trimethoprim + sulfamethoxazole)	Time dependent	T > MIC
Aminoglycosides (neomycin)	Concentration dependent	Cmax/MIC or AUC/MIC
Aminocyclitols (spectinomycin)		T > MIC
Polymyxins (colistin)	Concentration dependent	AUC/MIC

**Table 7:** Main antimicrobials used in swine for the treatment of enteric colibacillosis: routes of administration, dosages and main pharmacokinetic properties (modified from Burch [37])

Antimicrobial class/compounds	Administration and dosage (mg/kg body weight)			Pharmacokinetic Properties	Administration in enteric colibacillosis
	Injection	In water	In feed		
Trimethoprim/sulfonamide	15 (2.5 + 12.5)	30 (5 + 25)	15 (2.5 + 12.5)	Rapidly absorbed from intestine,well distributed in tissues;crosses uninflamed blood–brain barrier	IM and orally
Amoxicillin	7	20	15–20		IM and orally
Amoxicillin + Clavulanic acid	7 + 1,75				IM
Ceftiofur	3			Poorly absorbed from intestine,relatively poorly distributed intissues; crosses only inflamedblood–brain barrier	IM
Cefquinome	1–2				IM
Enrofloxacin	2.5			Well absorbed and distributed intissues	IM
Neomycin		11	11	Poorly absorbed from intestine,relatively poorly distributed intissues	Orally
Apramycin		7.5–12.5	4–8	Poorly absorbed from intestine,relatively poorly distributed intissues	Orally
Spectinomycin		10–50	1.1–2.2	Poorly absorbed from intestine,relatively poorly distributed intissues	Orally
Colistin sulphate		100,000 IU/Kg body weight	100,000 IU/Kg body weight	Not absorbed from intestine.	Orally

Three PK/PD indices have been proposed to allow predictions of antimicrobial efficacy: the ratio of the maximum unbound (free) concentration and the MIC (Cmax/MIC), the ratio of the free area under the concentration-time curve and the MIC (AUC/MIC), and the percentage of time thatthe concentration of free drug is above the MIC (T > MIC) [40].

Proper use of the PK/PD indices can optimize the dosage which, in turn, increase the possibility of therapeutic success, reduce toxicity and the emergence of resistance. To achieve a high probability of microbiological and clinical cure requires an adequate exposure of the bacteria to the antimicrobial agent. The exposure depends on the dose applied and is a function of different pharmacokinetic parameters such as drug bioavailability and clearance, as well as of the pathogen’s susceptibility (e.g. MIC). Interestingly, clinical breakpoints used for the MIC evaluation are calculated taking into account different criteria and are mainly influenced by PK/PD parameters [41].

For different antimicrobials, the approach based on pharmacokinetic parameters and MIC distribution are used to assess the potential of dose to treat a systemic infection by a pathogen. The distributions of MIC values for a pathogen are used to estimate the range of doses necessary to obtain a probability of therapeutic success according to the population pharmacokinetics data. Appropriate dosing of antibiotic is the key to control or clear bacteria on the site of infection but also to limit antimicrobial resistance. The PK/PD indices are mostly used as targets for efficacy in the process of dose selection, but it is also necessary to work on the dosage regimen optimization (frequency, length of the treatment) to reach the best clinical outcome and the lowest resistant bacteria selection [41].

##### Main characteristics of antimicrobials used in the treatment of enteric colibacillosis

ß-lactam antibiotics (such as penicillins, amoxicillin, cephalosporins, monobactams and carbapenem) prevent the bacterial cell wall from forming by interfering with the final stage of peptidoglycan synthesis. ß-lactam antibiotics act by binding to cell wall synthesis enzymes known as penicillin-binding proteins (PBPs), thereby inhibiting peptidoglycan synthesis. The drugs exert a bactericidal action, but cause lysis only of growing cells, that is, cells that are undergoing active cell-wall synthesis [42].

Amoxicillin achieves high tissue concentrations because it is well absorbed by the intestine. Amoxicillin, as most ß-lactams, is eliminated almost entirely by the kidneys, resulting in very high levels in the urine. Resistance to ß-lactams is mediated by efflux mechanisms and modification of porins in Gram negative bacteria that prevent the entry of the antibiotic [42].

Clavulanic acid is combined with amoxicillin in the ratio of 2:1 and the combinations are usually bactericidal. The combination has potential application in the treatment of a variety of infections in swine caused by plasmid-mediated ß-lactamase-producing bacteria, including neonatal diarrhoeal *E. coli*. Clavulanic acid is well absorbed after oral administration and has pharmacokinetic properties similar to amoxicillin [43].

Cephalosporins have the advantages of ß-lactamase stability, good activity against target proteins (PBPs), and good ability to penetrate bacterial cell walls. Fourth-generation cephalosporins are effective against Enterobacteriaecae and other Gram-negative bacteria resistant to earlier generations of cephalosporins because of acquired ß-lactamase-based resistance. Cephalosporins are among the safest antimicrobial drugs. The broad spectrum of antibacterial activity of second to fourth generation drugs may cause overgrowth of resistant bacteria including *Clostridium difficile*, which no longer have to compete with susceptible members of the microbial flora [44]. Ceftiofur sodium is available for use on swine in the treatment of neonatal colibacillosis. Fourth and third-generation cephalosporins are not first choice antimicrobial agents and should be reserved for use where susceptibility testing indicates that alternatives are not available. Narrower-spectrum drugs are often effective and should be preferred [44].

In general, the 3 mechanisms of ß-lactam resistance are: reduced access to the PBPs, reduced PBP binding affinity, and destruction of the antibiotic through the expression of ß-lactamase (enzymes that bind and hydrolyze ß-lactams) [45].

Antibacterial diaminopyrimidines are combined with a variety of sulfonamides (sulfadiazine, sulfamethoxazole, and sulfadoxine) in a fixed (1:5) ratio. The combination of a diaminopyrimidine with a sulfonamide inhibits sequential steps in the synthesis of folic acid and thus of the purines required for DNA bacterial synthesis. The interference by the diaminopyrimidine with recycling of tetrahydrofolic or dihydrofolic acid is probably responsible for the combination’s synergistic interaction. The combination has a wide margin of safety, and adverse effects can mainly be attributed to the sulfonamide. The diaminopyrimidine component is concentrated in tissues whereas the sulfonamide component moves only slowly from plasma into tissues. Trimethoprim-sulfonamide combinations have been used successfully in controlling a wide variety of conditions in pigs, including neonatal and post-weaning colibacillosis [46].

The aminoglycoside antibiotics kanamycin, gentamicin, amikacin, and neomycin are large molecules with numerous amino acid groups, making them basic polycations that are highly ionized at physiological pHs. The antibacterial action of the aminoglycosides is directed primarily against aerobic, Gram-negative bacteria. The bactericidal action of the aminoglycosides on aerobic Gram-negative bacteria is markedly influenced by pH, being most active in an alkaline environment. Increased local acidity secondary to tissue damage or bacterial destruction may explain the failure of aminoglycosides to kill usually susceptible pathogens. Aminoglycosides are poorly absorbed from the normal gastrointestinal tract, but are well absorbed after IM or SC injection. Elimination is entirely by renal excretion (glomerular filtration), and unchanged drug is rapidly excreted in the urine. Nephrotoxicity (acute tubular necrosis) is the most common adverse effect of aminoglycoside therapy. Most clinically important resistance to aminoglycosides is caused by plasmid-mediated enzymes, broadly classified as phosphotransferases, acetyltransferases, and adenyltransferases [47]. Gentamicin is used to treat neonatal colibacillosis in piglets from day 1 to day 3 of age, with either a single IM injection or an oral dose of 5 mg. [47].

Spectinomycin is an aminocyclitol antibiotic that lacks most of the toxic effects of the aminoglycoside antibiotics but, is limited in application by the ready development of resistance. Spectinomycin is a usually bacteriostatic, relatively broad-spectrum drug that can be bactericidal at concentrations 4 times the MIC. Chromosomal one-step mutation to high-level resistance develops readily in vivo and in vitro. Chromosomally resistant strains do not show cross-resistance with aminoglycosides. Pharmacokinetic properties are similar to those of the aminoglycosides. In pigs, spectinomycin is available as an oral solution for the treatment of colibacillosis [47].

After administration, fluoroquinolones exhibit rapid and extensive tissue distribution because of their hydrophilic nature and low (<50%) protein binding. High concentrations are found in the bile and organs of excretion (liver, intestine, and urinary tract). The fluoroquinolones are predominantly excreted as unchanged drug in the urine by glomerular filtration and active tubular secretion. Fluoroquinolones are relatively safe antimicrobial drugs. Administered at therapeutic doses, toxic effects are mild and generally limited to gastrointestinal disturbances such as nausea, vomiting, and diarrhoea. Resistance to fluoroquinolones occurs by target modification, decreased permeability, efflux and/or target protection. Each of these fluoroquinolone resistance mechanisms can occur simultaneously within the same cell, thereby leading to very high resistance levels [48]. Fourth and third generation cephalosporins and fluoroquinolones are not first choice antimicrobial agents and should be reserved for use where susceptibility testing indicates that alternatives are not available.

Colistin (polymyxin E) is a cationic, multicomponent, lipopeptide antibacterial agent discovered soon after the end of the Second World War (1949). Colistin is a bactericidal drug that binds to lipopolysaccharide (LPS) and phospholipids in the outer membrane of Gram negative bacteria. This process results in an increase in the permeability of the cell envelope, leakage of cell contents, and subsequent cell death. Colistin sulphate is the only approved product in some countries for oral use in pig production, to control intestinal infections caused by Enterobacteriaceae [49]. Polymyxins are well tolerated after oral or local administration, but systemic use causes nephrotoxic, neurotoxic, and neuromuscular blocking effects.

The use of colistin in Europe varies widely between countries. Countries with intensive livestock production can have a level of usage below 1 mg/PCU (e.g. Denmark and the UK) or much higher, up to 20 to 25 mg/PCU (Italy and Spain) [50]. Due to its importance in human medicine, the public health impact of current or future use of colistin products in animals has been under discussion for long time [49]. The usually recommended dose for therapy is 100,000 IU/kg BW (100,000 IU/kg body weight per day or 50,000 IU/kg administered at 12-h intervals), but in some non-European countries the use of colistin is authorised at lower dosage, as feed additive for growth promotion [51].

##### *E.coli* and antimicrobial resistance

Antimicrobial resistance to several antibiotics such as apramycin, neomycin, trimethoprim-sulphametoxazole and colistin has been increasingly observed in ETEC strains causing PWD [3]. The development of resistance to a wide range of antimicrobial drugs, as well as the demonstrated trend of resistance in ETEC strains to the antibiotics used for the treatment of colibacillosis in pigs, is nowadays a reason for concern [52]. Multidrug resistance among ETEC isolates has been described and recently there has been an increasing tendency for porcine ETEC to express a multidrug-resistant phenotype [33].

It is difficult, if not impossible, to provide general data on resistance, because the situation is variable in different countries and pig populations, and mainly depends on the antimicrobials preferentially used. Table 8 reports the data of resistance of *E.coli* strains to different antibiotics commonly used in the treatment of colibacilloisis.

**Table 8:** Examples of resistance rates to different antibiotics of *E.coli* strains isolated from healthy and diseased pigs in different countries (Modified from Aarestrup et al. 2008) [33]

ANTIBIOTIC	USA^a^	Brazil^b^	Korea^c^	China^d^	Spain^e^	Belgium^e^	Germany^e^	France^e^	Poland^f^
Fluoroquinolones	0%	30%	64.9%	64%	14%	39%	8%	6%	30%
Ceftiofur	22%	-	-	-	4%	1%	1%	1%	-
SXT	23%	62%	75.7%	90%	-	71%	51%	66%	78.8%
Neomycin	66%	32.8%	-	9.4%	20%	2%	-	11%	-
Apramycin	30%	-	-	-	13%	13%	10%	3%	-
Gentamicin	48%	39%	77%	57%	20%	46%	12%	6%	45%
REFERENCE	[59]	[53]	[57]	[60]	[33]	[33]	[33]	[33]	[61]

^a^(*E.coli* isolated form pigs with diarrhoea and septicemia); ^b^(E.coli isolated from cases of neonatal colabacillosis; ^c^(*E.coli* isolated from pigs with diarrhoea); ^d^(pathogenic and commensal *E.coli* isolated from pigs); ^e^(*E.coli* isolated from diseased pigs); ^f^(commensal *E.coli*)

In a study performed in Italy, aiming to evaluate the trend of resistance of ETEC isolated in a 10-year period (2002–2011), 442 strains of F4-positive *E.coli* isolated from cases of porst-weaning colibacillosis were tested against several antibiotics using the disc diffusion method [52]. In the study, intermediate strains were grouped with the resistant one. Isolates showed a statistically significant increasing trend of resistance over the whole period of study to: enrofloxacin (from 14.5% to 89.3%), flumequine (from 49.1% to 92.9%) and cefquinome (from 3.8% to 44%). An increasing resistance (not statistically significant) was also observed to gentamicin (from 63.6% to 85.7%), apramycin (from 61.8% to 82.1%), and trimethoprim-sulphametoxazole (from 75% to 89.3%).

Resistance to enrofloxacin was described in *E. coli* strains isolated in Brazil, where nearly 30% of the isolates from cases of neonatal colibacillosis were resistant to this antibiotic [53]. Fluorochinolones resistance has been strongly correlated with the quantity of the drug used to treat pigs and plasmid-borne transfer of fluorochinolones resistance has been demonstrated in pig *E. coli* strains [54].

High levels of resistance to gentamicin were reported in *E. coli* isolated form diseased pigs in Belgium (46%), Poland (45%) and Spain (20%) [33]. Resistance to gentamicin and other aminoglycosides is usually transmissible and cross-resistance between gentamicin and apramycin was described [55].

The *E. coli* isolates from cases of PWD in Australia resulted resistant to streptomycin spectinomycin, ampicillin and trimethoprim-sulphametoxazole. In the same study, a smaller number of isolates were resistant to neomycin and apramycin, and a proportion of these showed resistance to gentamicin. None of the isolates were resistant to enrofloxacin or ceftiofur [56].

A study performed in Korea showed how *E. coli* strains isolated from diarrhoeic pigs were multi-resistant (resistant to more than 4 antibiotics) with high levels of resistance to several antibiotics: gentamicin (77%), trimethoprim-sulphametoxazole (75.7%), amoxicillin (75.7%), ampicillin (73%) and enrofloxacin (64.9%) [57].

In the last few years, *E. coli* strains resistant to colistin has become more common. Strains of *E. coli* with acquired resistance are encountered among pathogenic isolates, commonly in pigs suffering from diarrhoea [51] (Table 9).

**Table 9:** Examples of colistin resistance in *E.coli* strains isolated from healthy and diseased pigs in different countries (Modified from Kempf et al. [51])

Country	Origin of the isolates	% of resistance/non-wild type strains	Reference
France	faeces, healthy pigs	0.5%	[62]
Sweden	healthy pigs	0%	[63]
Denmark	healthy pigs	0%	[64]
Belgium	pigs with diarrhoea	9.6%	[65]
Croatia	pigs with diarrhoea	3%	[66]
Brazil	pigs with diarrhoea	28.1%	[53]
UK	Slaughterhouse, healthy pigs	34,1%	[67]
China	pigs with diarrhoea	33.3%	[68]

Resistance to colistin is based on mutations responsible for modification of the LPS charge. Until now, polymyxin resistance has involved chromosomal mutations making the resistance mechanism unstable and incapable of spreading to other bacteria but has never been reported via horizontal gene transfer. A study performed in China on antimicrobial resistance in commensal *E. coli* from food animals has shown an increase of colistin resistance and has described the emergence of the first transmissible, plasmid-mediated polymyxin resistance in the form of *mcr*-1 [58]. The gene can be easily transferred between different types of bacteria, potentially leading to rapid development of resistance. While the gene was first detected in *E.coli* in China, it has subsequently also been found in the EU. In terms of antibiotic resistance, plasmids play a central role as vehicles for resistance gene capture and subsequent dissemination.

The European Commission, following the recent discovery of this new mechanism of resistance in bacteria to colistin (caused by the *mcr*-1 gene), requested an update from the EMA’s Antimicrobial Advice Ad Hoc Expert Group (AMEG) on its 2013 advice on the “use of colistin products in animals within the European Union”. In its advice published in July 2016, the Expert Group describes several measures that should be considered to tackle the problem. These are summarised as follow:

- Over the course of the next 3–4 years, all Member States should reduce the use of colistin in animals at least to a target level of 5 mg colistin/PCU.
- Member States are also encouraged to set stricter national targets, ideally below 1 mg colistin/PCU as a desirable level.
- The reduction of colistin sales should not be compensated for by an increase in the use of other types of antimicrobials, but should be achieved through other measures such as improved farming conditions, biosecurity between production cycles, and vaccination of livestock.
- Colistin should be reclassified and added to Category 2 of the AMEG classification system, which includes medicines reserved for treating infections in animals for which no effective alternative treatments exist [50].

## Conclusion

The control of a disease begins with a correct diagnostic approach. The diagnosis of colibacillosis require an appropriate sampling for isolation of the pathogen and standardized diagnostic criteria, including the evaluation of antimicrobial susceptibility.

The therapeutic use of antimicrobials is widely practiced to control both neonatal and post-weaning colibacillosis, even if in many countries the prophylactic and metaphylactic use of the antibiotics is still common. Growing concern on the increase of antimicrobial resistance among pathogenic *E. coli* strains with an increased prevalence of multi-resistant *E. coli* strains from diarrhoeic pigs is leading to more attention on the alternatives to antibiotics such as vaccines, probiotics, prebiotics, additives, and management practices. Even if some preventive approaches have shown some promise and efficacy, in many cases the use of antibiotics is preferred to treat and control enteric colibacillosis.

Nowadays, there is concern over the increased phenomenon of antimicrobial resistance among bacteria isolated from production animals. The risk linked to this phenomenon increases if antimicrobials are used inappropriately, for example, in an untargeted manner (e.g. mass medication or use on non-susceptible microorganisms), at sub-therapeutic doses, repeatedly, or for inappropriate periods of time. These conditions force a more rational and judicious use of antibiotics.

## Abbreviations

AIDA: Adhesin involved in diffuse adherence
AMEG: Antimicrobial Advice Ad Hoc Expert Group
AUC: Area under the plasma concentration time curve
BW: Body weight
cGMP: Cyclic guanosine monophosphate
Cmax: Maximum plasma concentration
EAST1: Enteroaggregative *E. coli* heat stable enterotoxin
ED: Oedema disease
EMA: European Medicines Agency
EPEC: Enteropathogenic *E.coli*
ETEC: Enterotoxigenic *Escherichia coli*
IM: Intramuscular
LPS: Lipopolysaccharide
LT: Heat labile toxin
MIC: Minimum inhibitory concentration
PBPs: Penicillin-binding protein
PCR: Polymerase chain reaction
PCU: Population correction unit
PD: Pharmacodynamics
PEDV: Porcine epidemic diarrhoea virus
PK: Pharmacokinetics
PWD: Post-weaning diarrhoea
qPCR: Quantitative PCR
SC: Subcutaneous
STa: Heat stable toxin a
STb: Heat stable toxin b
STEC: Shiga toxin *E.coli*
Stx2e: Shiga toxin
T: Time
TGEV: Transmissible gastro enteritis virus

## References

[1] Fairbrother JM, Gyles CL, Zimmerman JJ, Karriker LA, Ramirez A, Schwartz KJ, Stevenson GW (2012). Colibacillosis. Disease of Swine.

[2] Sjölund M, Zoric M, Wallgren P. Financial impact on pig production: III. Gastrointestinal disorders: Proceedings of the 6th European Symposium of Porcine Health Management, Sorrento; 2014. p. 189–Italy.

[3] Zhang W (2014). Progress and Challenges in Vaccine development against enterotoxigenic *Escherichia coli* (ETEC) – Associated porcine Post-weaning Diarrhea (PWD). J Vet Med Res.

[4] Burrow E, Simoneit C, Tenhaggen BA, Käsbohrer A (2014). Oral antimicrobial resistance in porcine *E. coli* – A systematic review. Prev Vet Med.

[5] Lorenz I (2009). D-Lactic acidosis in calves. Vet J.

[6] Melkebeek V, Goddeeris BM, Cox E (2013). ETEC vaccination in pigs. Vet Immunol Immunopathol.

[7] Francis DH (2002). Enterotoxigenic *Escherichia coli* infection in pigs and its diagnosis. J Swine Health Prod.

[8] Ravi M, Ngeleka M, Kim SH, Gyles C, Berthiaume F, Mourez M (2007). Contribution of AIDA-I to the pathogenicity of a porcine diarrheagenic *Escherichia coli* and to intestinal colonization through biofilm formation in pigs. Vet Microbiol.

[9] Zajacova ZS, Faldyna M, Kulich P, Kummer V, Maskova J, Alexa P (2013). Experimental infection of gnotobiotic piglets with *Escherichia coli* strains positive for EAST1and AIDA. Vet Immunol Immunopathol.

[10] Dubreuil JD, Isaacson RE, Schifferli DM. Animal Enterotoxigenic *Escherichia coli*. EcoSal Plus. 2016; doi:10.1128/ecosalplus.ESP-0006-2016. 10.1128/ecosalplus.esp-0006-2016 PMC5123703 27735786

[11] Johnson AM, Kaushik RS, Francis DH, Fleckenstein JM, Hardwidge PR (2009). Heat-Labile Enterotoxin Promotes *Escherichia coli* Adherence to Intestinal Epithelial Cells. J Bacteriol.

[12] Dou S, Gadonna-Widehem P, Rome V, Dounia Hamoudi D, Thibaut Larcher T, Bahi-Jaber N, Pinon-Quintana A, Guyonvarch A, Huërou-Luron ILE, Abdennebi-Najar L. Characterisation of Early-Life Fecal Microbiota in Susceptible and Healthy Pigs to Post-Weaning Diarrhoea. PLoS One. 2017; doi: 10.1371/journal.pone.0169851. 10.1371/journal.pone.0169851 PMC5225014 28072880

[13] Pluske JR, Pethick DW, Hopwood DE, Hampson DJ (2002). Nutritional influences on some major enteric bacterial diseases of pigs. Nutr Res Rev.

[14] Katouli M, Lund A, Wallgren P, Kühn I, Söderlind O, Möllby R (1995). Phenotypic characterization of intestinal *Escherichia coli* of pigs during suckling, postweaning, and fattening periods. Appl Environ Microbiol.

[15] Marchant M, Moreno MA (2013). Dynamics and Diversity of *Escherichia coli* in Animals and System Management of the Manure on a Commercial Farrow-to-Finish Pig Farm. Appl Environ Microbiol.

[16] Laine TM, Lyytikäinen T, Yliaho M, Anttila M (2008). Risk factors for post-weaning diarrhoea on piglet producing farms in Finland. Acta Vet Scand.

[17] Moredo FA, Piñeyro PE, Márquez GC, Sanz M, Colello R, Etcheverría A (2015). Enterotoxigenic *Escherichia coli* Subclinical Infection in Pigs: Bacteriological and Genotypic Characterization and Antimicrobial Resistance Profiles. Foodborne Pathog Dis.

[18] Osek J (1999). Prevalence of virulence factors of *Escherichia coli* strains isolated from diarrheic and healthy piglets after weaning. Vet Microbiol.

[19] Lawley TD, Walker AW (2013). Intestinal colonization resistance. Immunology.

[20] Kim M, Ashida H, Ogawa M, Yoshikawa Y, Mimuro H, Sasakawa C (2010). Bacterial interactions with the host epithelium. Cell Host Microbe.

[21] Hampson DJ, Fu ZF, Bettleheim KA, Wilson MW (1988). Managemental influences on the selective proliferation of two strains of haemolytic *Escherichia coli* in weaned pigs. Epidemiol Infect.

[22] Fairbrother JM, Nadeau É, Gyles CL (2005). *Escherichia coli* in postweaning diarrhea in pigs: an update on bacterial types, pathogenesis, and prevention strategies. Anim Health Res Rev.

[23] Luppi A, Gibellini MV, Gin T, Vangroenweghe F, Vandenbroucke V, Bauerfeind R (2016). Prevalence of virulence factors in enterotoxigenic *Escherichia coli* isolated from pigs with post-weaning diarrhoea in Europe. Porcine Health Manag.

[24] Casey TA, Bosworth BT (2009). Design and evaluation of a multiplex polymerase chain reaction assay for the simultaneous identification of genes for nine different virulence factors associated with *Escherichia coli* that cause diarrhea and edema disease in swine. J Vet Diagn Investig.

[25] Vazquez-Marrufo G, Rosales-Castillo JA, Robinson-Fuentes VA, Tafolla-Munoz I, Carreras-Villase N, Vazquez-Garcidue MS (2017). Multi-Typing of Enterobacteria Harboring LT and ST Enterotoxin Genes Isolated from Mexican Children. Jpn J Infect Dis.

[26] Ouyang-Latimer J, Ajami NJ, Jiang ZD, Okhuysen PC, Paredes M, Flores J (2010). Biochemical and genetic diversity of enterotoxigenic *Escherichia coli* associated with diarrhea in United States students in Cuernavaca and Guadalajara, Mexico, 2004–2007. J Infect Dis.

[27] Ståhl M, Kokotovic B, Hjulsager CK, Breum SØ, Angen Ø (2011). The use of quantitative PCR for identification and quantification of *Brachyspira pilosicoli*, *Lawsonia intracellularis* and *Escherichia coli* fimbrial types F4 and F18 in pig feces. Vet Microbiol.

[28] Martelli P. Tabelle diagnosi differenziale. In: Martelli P, editor. “Le patologie del maiale”. Point Veterinaire Italie Editor; 2013. p. 2–5.

[29] Biwater RJ (1983). Diarrhoea treatments, fluid replacement and alternatives. Ann Rech Vet.

[30] Thomson J, Friendship RM, Zimmerman JJ, Karriker LA, Ramirez A, Schwartz KJ, Stevenson GW (2012). Digestive System. Disease of Swine.

[31] Roselli M, Finamore A, Garaguso I, Britti MS, Mengheri E (2003). Zinc oxide protects cultured enterocytes from the damage induced by *Escherichia coli*. J Nutr.

[32] Yazdankhah S, Rudi K, Bernhoft A Zinc and copper in animal feed - development of resistance and co-resistance to antimicrobial agents in bacteria of animal origin. Microb Ecol Health Dis. 2014. doi: 10.3402/mehd.v25.25862. 10.3402/mehd.v25.25862 PMC4179321 25317117

[33] Aarestrup FM, Oliver Duran C, Burch DG (2008). Antimicrobial resistance in swine production. Anim Health Res Rev.

[34] Vahjen W, Pietruszyńska D, Starke IC, Zentek J (2015). High dietary zinc supplementation increases the occurrence of tetracycline and sulfonamide resistance genes in the intestine of weaned pigs. Gut Pathog.

[35] CVMP opinions on veterinary medicinal products. Committee for Medicinal Products for Veterinary Use (CVMP) Meeting of 14–16 March 2017 EMA/CVMP/147249/2017. European Medicines Agency. Press release. http://www.ema.europa.eu/.

[36] DGS B, Giguère S, Prescott JF, Dowling PM (2013). Antimicrobial Drug use in swine. Antimicrobial Therapy in Veterinary Medicine.

[37] Burch DGS, Duran OC, Aarestrup FM, Guardabassi L, Jensen LB, Kruse H (2008). Guidlines for antimicrobial use of in swine. Guide to Antimicrobial Use in Animals.

[38] COMMISSION NOTICE. Guidelines for the prudent use of antimicrobials in veterinary medicine (2015/C 299/04). Off J Eur Union. Available at http://www.ema.europa.eu/docs/en_GB/document_library/Report/2009/11/WC500008770.pdf

[39] Martinez MN, Toutain PL, Turnidge J, Giguère S, Prescott JF, Dowling PM (2013). The Pharmacodynamics of Antimicrobial Agents. Antimicrobial Therapy in Veterinary Medicine.

[40] Page SW, Gautier P (2012). Use of antimicrobial agents in livestock. Rev Sci Tech Off Int Epiz.

[41] Ahmad I, Huang L, Hao H, Sanders P, Yuan Z. Application of PK/PD Modeling in Veterinary Field: Dose Optimization and Drug Resistance Prediction. BioMed Res Int. 2016. 10.1155/2016/5465678 10.1155/2016/5465678 PMC4771886 26989688

[42] Prescott JF, Giguère S, Prescott JF, Dowling PM (2013). Beta-lactam Antibiotics: Penam Penicillins. Antimicrobial Therapy in Veterinary Medicine.

[43] Prescott JF, Giguère S, Prescott JF, Dowling PM (2013). Beta-lactam Antibiotics: Beta-lactamase Inhibitors, Carbapenems, and Monobactams. Antimicrobial Therapy in Veterinary Medicine.

[44] Prescott JF, Giguère S, Prescott JF, Dowling PM (2013). Beta-lactam Antibiotics: Cephalosporins. Antimicrobial Therapy in Veterinary Medicine.

[45] Rice LB (2012). Mechanisms of Resistance and Clinical Relevance of Resistance to ß-Lactams, Glycopeptides, and Fluoroquinolones. Mayo Clin Proc.

[46] Prescott JF, Giguère S, Prescott JF, Dowling PM (2013). Sulfonamides, Diaminopyrimidines, and Their Combinations. Antimicrobial Therapy in Veterinary Medicine.

[47] Dowling PM, Giguère S, Prescott JF, Dowling PM (2013). Aminoglycosides and Aminocyclitols. Antimicrobial Therapy in Veterinary Medicine.

[48] Giguère S, Dowling PM, Giguère S, Prescott JF, Dowling PM (2013). Fluoroquinolones. Antimicrobial Therapy in Veterinary Medicine.

[49] Rhouma M, Beaudry F, Letellier A (2016). Resistance to colistin: what is the fate for this antibiotic in pig production?. Int J Antimicrb Agents.

[50] European Medicines Agency: Updated advice on the use of colistin products in animals within the European Union: development of resistance and possible impact on human and animal health. 2016. http://www.ema.europa.eu/docs/en_GB/document_library/Scientific_guideline/2016/07/WC500211080.pdf

[51] Kempf I, Fleury MA, Drider D, Bruneau M, Sanders P, Chauvin C (2013). What do we know about resistance to colistin in Enterobacteriaceae in avian and pig production in Europe?. Int J Antimicrob Agents.

[52] Luppi A, Bonilauri P, Dottori M, Gherpelli Y, Biasi G, Merialdi G (2015). Antimicrobial resistance of F4+ *Escherichia coli* isolated from Swine in Italy. Transbound Emerg Dis.

[53] Costa MM, Drescher G, Maboni F, Weber SS, Schrank A, Vainstein MH (2010). Virulence factors, antimicrobial resi stance, and plasmid content of *Escherichia coli* isolated in swine commercial farms. Arg Bras Med Vet Zootec.

[54] Barton MD (2014). Impact of antibiotic use in the swine industry. Curr Opin Microbiol.

[55] Jensen VF, Jakobsen L, Emborg HD (2006). Correlation between apramycin and gentamicin use in pigs and an increasing reservoir of gentamicin-resistant *Escherichia coli*. J Antimicrob Chemother.

[56] Smith MG, Jordan D, Chapman TA, Chin JJ, Barton MD, Do TN (2010). Antimicrobial resistance and virulence gene profiles in multi-drug resistant enterotoxigenic *Escherichia coli* isolated from pigs with post-weaning diarrhoea. Vet Microbiol.

[57] Lee SI, Rayamahji N, Lee WJ, Cha SB, Shin MK, Roh YM (2009). Genotypes, antibiogram, and pulsed-field gel electrophoresis profiles of *Escherichia coli* strains from piglets in Korea. J Vet Diagn Investig.

[58] Liu YY, Wang Y, Walsh TR, Yi LX, Zhang R, Spencer J (2016). Emergence of plasmid-mediated colistin resistance mechanism MCR-1 in animals and human beings in China: a microbiological and molecular biological study. Lancet Infect Dis.

[59] Malik YS, Chander Y, Olsen K, Goyal SM (2011). Antimicrobial resistance in enteric pathogens isolated from Minnesota pigs from 1995 to 2004. Can J Vet Res.

[60] Jiang HX, Lü DH, Chen ZL, Wang XM, Chen JR, Liu YH (2011). High prevalence and widespread distribution of multi-resistant *Escherichia coli* isolates in pigs and poultry in China. Vet J.

[61] Mazurek J, Bok E, Stosik M, Baldy-Chudzik K (2015). Antimicrobial resistance in commensal *Escherichia coli* from pigs during metaphylactic trimethoprim and sulfamethoxazole treatment and in the post-exposure period. Int J Environ Res Public Health.

[62] Belloc C, Nam Lam D, Laval A (2008). Low occurrence of colistin-resistant *Escherichia coli* in faecal content of pigs in French commercial herds. Revuede Med Vet.

[63] Statens Veterinärmedicinska Anstalt (SVA). SVARM 2010. Swedish veterinary antimicrobial resistance monitoring. SVA. 2011. http://www.sva.se/globalassets/redesign2011/pdf/om_sva/publikationer/trycksaker/svarm2010.pdf.

[64] Statens Serum Institut; Danish Veterinary and Food Administration; DanishMedicines Agency; National Veterinary Institute; Technical University of Denmark; National Food Institute. DANMAP 2009—Use of antimicrobial agents and occurrence of antimicrobial resistance in bacteria from food animals, foods and humans in Denmark. 2010. http://orbit.dtu.dk/files/6329669/Danmap+2010.pdf.

[65] Boyen F, Vangroenweghe F, Butaye P, De Graef E, Castryck F, Heylen P (2010). Disk prediffusion is a reliable method for testing colistin susceptibility in porcine *E. coli* strains. Vet Microbiol.

[66] Habrun B, Dragica S, Kompes G, Benic M (2011). Antimicrobial susceptibility of entero-toxigenic strains of *Escherichia coli* isolated from weaned pigs in Croatia. Acta Vet.

[67] Enne VI, Cassar C, Sprigings K, Woodward MJ, Bennett PM (2008). A high prevalence of antimicrobial resistant *Escherichia coli* isolated from pigs and a low preva-lence of antimicrobial resistant *E. coli* from cattle and sheep in Great Britain at slaughter. FEMS Microbiol Lett.

[68] Lu L, Dai L, Wang Y, Wu C, Chen X, Li L (2010). Characterization of antimicrobial resistance and integrons among *Escherichia coli* isolated from animal farms in Eastern China. Acta Trop.

